# Diagnosis and Course of Moon-blindness in Army Horses

**Published:** 1897-08

**Authors:** 


Diagnosis and Course of Moon-blindness in Army Horses.—Dr. Nowotny, in the *Thierärztliche Centralblatt*, November 15, 1896, takes exception to the statement of Dr. Kirnbauer, in Arad, that the chief responsibility for the frequency of this disease falls upon the veterinarians who have not examined the eyes of all the recruiting horses with the ophthalmoscope, or do not understand how to use the instrument. He feels it his duty to communicate a few observations made in another land under other circumstances, so that the natural conclusion to Kirnbauer's publication may not be too hastily generalized.

The Fifth Dragoon Regiment, stationed at four places in Styria, gets the largest part of its recruiting horses from Hungary, and since the disease mentioned occurs less frequently in these horses some influence of the climate and altitude of the land upon the animals must be admitted.

According to the statistics of 1894 there were five horses (5 per cent.) in 1895, three horses (3 per cent.) affected with internal periodic inflammation of the eyes. In the two years preceding the number of horses that became moon-blind certainly did not exceed 10 per cent. annually. As will appear later, it cannot be asserted that any special service is to be ascribed to the examining veterinarian. He has examined with the ophthalmoscope all the horses belonging to the two squadrons of dragoons stationed at Graz and Marberg, as well as all the recruiting horses during the last two years, and made notes in regard to the smallest ocular defects.

Dr. Nowotny treats, first, of the traumatic and then the external periodic inflammation of the eye at the period of inflammation on the one hand, and, on the other, he deals with the defects present at birth or occurring otherwise, as well as the changes which are superinduced by traumatic or symptomatic inflammation of the eye, but which are noticed at a time when inflammation is not present.

**It has within its borders the homes of some three Presidents of our country.**

The destruction of the pupil of the eye, with a decrease in the intraocular compression, is to be regarded as the pathognomonic symptom of an attack of moon-blindness which has passed away. This symptom is perceptible to the naked eye and to the touch. If these symptoms are not marked, which is not seldom after the first attack, then the ophthalmoscope gives the best means of reaching a diagnosis.

Dr. Nowotny then cites several cases of traumatic inflammation and external periodic inflammation which he had observed. In some cases there were no marks left behind, in others small pigment spots were left on the front lens, or the cornea showed marks of injury.

However, he asserts that muddy places on the front and less frequently on the back lens are often found, which are to be regarded as pigment spots, but not the product of an inflammation of the uvea or the consequence of a traumatic inflammation. They are merely anomalies in the pigment. He cites some thirty-one horses in which these had been noticed, and in no one of them did the animal suffer from eye-trouble. The anomaly can be referred to causes of a purely local nature, as a little portion of the uveal pigment can at times become loose and become attached at one point to the lens-capsule. In reference to moon-blindness proper, seventeen cases are cited, which he had observed from the time of the first attack. In some cases only one eye was attacked, in others both. The period between the first and second attacks varied greatly, and in many cases blindness of one or both eyes followed ultimately. Not always after the first attack did such changes in the affected eye remain as could be detected without the help of the ophthalmoscope, and in not a few cases such unimportant changes have taken place that the examination with this instrument is practically without result. The color of the pigment varies, and the changes are in many cases so small that even a veterinarian experienced in the use of the instrument will reach no definite result. In most cases the investigation of the transparent media of the eye must be made in ordinary daylight, and the examination of the background of the eye will only in rare cases show such changes as can be regarded as pathognomonic symptoms of moon-blindness.

**Remember the Southern field is just opening.**

Most horses become moon-blind between three and six years of age, although the first attack may not take place until the animal is between eleven and fifteen years old. Two fresh horses which he examined with the ophthalmoscope in the spring of last year and accepted as sound were attacked very soon after that,—the first one in thirty-eight days, the second in seven weeks—so that we have a proof that horses which have been found all right by the examiner can shortly after become a prey to moon-blindness, the minute inclination to this trouble not always being detectable with the ophthalmoscope.

From his observations Nowotny draws the following conclusions: 1. That in spite of examination with the ophthalmoscope by a qualified veterinarian many cases of internal periodical inflammation will occur in fresh horses in the future. 2. As the law regarding the purchase of military horses now stands, an attack of moon-blindness in the future can only be regarded in the light of a defect which only partially falls under the conditions of the law. 3. That a too severe procedure on the part of the examining veterinarian will lead to the rejection of many good horses, as slight local anomalies, as has been explained, often occur and lead to no further trouble.

In conclusion, it should be remembered that many military horses which have been mustered out are sold and used for breeding purposes. As long as these animals have healthy eyes no evil can come of it, but it would be a wise provision if those with moon-blindness were so branded, so that no deception can be practised and the owner claim that the animal went blind as the result of some injury.

**It will have places for your brothers and your children in the future.**

